# Reflecting on activities which support public involvement within an evaluation of public involvement reports from facilities funded by the national institute for health and care research: a co-produced commentary

**DOI:** 10.1186/s40900-024-00579-x

**Published:** 2024-05-10

**Authors:** Alice Moult, Ali Aries, Paul Bailey, Zoe Paskins

**Affiliations:** 1 Impact Accelerator Unit, Keele University, Newcastle-under-Lyme, ST5 5BG, 0000-0002, 9424-5660 UK; 2 School of Allied Health, Keele University, Newcastle-under-Lyme, ST5 5BG UK; 3 School of Medicine, Keele University, Newcastle-under-Lyme, ST5 5BG UK; 4 Haywood Academic Rheumatology Centre, Haywood Hospital, Midland Partnership University NHS Foundation Trust, Stafford, ST5 5BG UK

**Keywords:** Patient and public involvement and engagement, Review, Commentary, Co-production

## Abstract

Although including public contributors as members of research teams is becoming common, there are few reflections on how they have been incorporated, and almost none of these reflections are co-produced with public contributors. This commentary, written by both academics and a public contributor, reflects on Patient and Public Involvement (PPI) activities when undertaking a framework analysis of PPI sections of annual reports from the National Institute for Health and care Research (NIHR) funded research centres. The UK Standards for Public Involvement (inclusive opportunities, working together, support and learning, communications, impact and governance) were used to structure our reflections. Key topics of reflection were: how difficult it is, in practice, to incorporate PPI into all aspects of the research cycle, especially when completing a commissioned research project on a short time-frame, and the complexities of incorporating PPI into qualitative analysis. Although useful when reflecting upon our own PPI practices, ways in which the UK Standards for Public Involvement could be improved were suggested. We hope that the co-produced recommendations can be used by other teams engaging with public contributors.

## Abstract (plain language summary)

Although including public contributors as members of research teams is becoming common, there are few reflections on how they have been incorporated, and almost none of these reflections are co-produced with public contributors. This commentary, written by both academics and a public contributor, reflects on Patient and Public Involvement (PPI) activities when undertaking an evaluation of PPI sections of annual reports from the National Institute for Health and care Research (NIHR) funded research centres. The UK Standards for Public Involvement (inclusive opportunities, working together, support and learning, communications, impact and governance) were used to structure our reflections. Key topics of reflection were: how difficult it is, in practice, to incorporate PPI into all aspects of the research cycle, especially when completing a commissioned research project within a short time-frame, and the complexities of incorporating PPI into qualitative analysis. Although useful when reflecting upon our own PPI practices, ways in which the UK Standards for Public Involvement could be improved were suggested. We hope that the co-produced recommendations can be used by other teams engaging with public contributors.

## Background

Whilst evidence suggests that Patient and Public Involvement (PPI) can improve health research, there is a lack of guidance in how to involve the public in an effective way [1]. Research suggests that a gap remains in understanding how PPI influences research and proposes that the solution is providing more detailed accounts of PPI [2, 3]. There is an emerging evidence base documenting the various approaches to PPI in research [4], such literature encourages reflective practices that can help to share PPI activities that work in certain research contexts. Evaluation frameworks for PPI have been criticised because the methods give precedence to indicators that might matter to researchers, not the public [5]. These frameworks often examine a one-way exchange of information that does not capture the reciprocal learning between researchers and the public.

This co-produced commentary is underpinned by Knowles’ [6] call for comparative examples which explore differences in PPI approaches in different research contexts and Staley and Barron’s [7] conceptualisation of PPI as conversations between public contributors and researchers which support mutual learning. The commentary, written by both academics and a public contributor, reflects on PPI activities when conducting a commissioned piece of work evaluating the public involvement sections of annual reports from the National Institute for Health and Care Research (NIHR) funded research centres [8]. From these reflections, we co-produced recommendations for researchers and research commissioners when involving public contributors in a commissioned project with a short-time frame. We hope this commentary will encourage the sharing of learning and promote best PPI practice.

### The commissioned project

The project was led by Alice Moult (AM - Research Fellow in Knowledge Mobilisation). Other researchers included a Research Assistant in Applied Health, a senior Lecturer in Physiotherapy (AA), a Lecturer in Mental Health and Wellbeing, an Associate Professor in PPI and a Reader and Honorary Consultant in Rheumatology. Paul Bailey was a public contributor. PB’s vocation was as a Prison Officer/Tutor.

Within the United Kingdom (UK) the NIHR is the largest funder of health and social care research. The NIHR also funds research centres (e.g. Biomedical Research Centres, Applied Research Centres) that support the delivery of research studies. Each year, award-holders (those who are funded by the research centres) are required to write a report describing their activities. These reports included PPI activities. The overall aim of commissioned project was to evaluate the breadth and quality of PPI as reported by NIHR research centres. The project’s time-frame was six months (June 2022 – December 2022).

The project analysed 112 reports using the six UK Standards for Public Involvement (henceforth referred to as the UK Standards): inclusive opportunities, working together, support and learning, communications, impact and governance [9]. A quality improvement framework named Insights [10] was used to separate PPI practice into one of four levels of increasing quality: ‘Welcoming’, ‘Listening’, ‘Learning’ and ‘Leading’.

The findings suggested that PPI activities, of varying quality, covered all six UK Standards. A number of award-holders either planned to, or had begun, working towards increasing the diversity of public contributors within their research. Methods of working with public contributors were varied. Most award-holders offered support and learning opportunities for both PPI members (e.g. ‘taster’ PPI sessions or peer support) and researchers (e.g. blogs, videos, podcasts). Some award-holders invited PPI members to co-produce communication plans relating to study materials and research findings. The impact of public involvement was described in terms of benefits to PPI members themselves, and on project and award-holder levels. Many award-holders reported inviting public contributors to share decision-making within and about governance structures. The Insights framework was useful in determining the quality of PPI activities relating to each UK Standard. Recommendations for improving the quality of future PPI activities were co-developed with stakeholders and public contributors. A detailed description of the study can be found elsewhere [8].

### How were public contributors involved in the commissioned project?

The researchers convened a PPI group (two members of Keele Medical Schools Research and User Group (RUG) and an individual aligned to the School of Allied Health, Keele University [PB]) specifically for this study.

Keele University has a long track record in PPI in research, with over 160 RUG members at any one time. RUG members are currently contributing to 64 projects across all aspects of the research cycle. These public contributors advise on development of research questions, co-design grant applications, work as co-applicants, advise on research advisory groups, and share lived experience expertise at Trial Steering Committee meetings, Study Advisory Group meetings and research team project meetings. Keele University’s Impact Accelerator Unit (IAU) hosts all PPI in research for the School of Medicine and has a team of four professional services staff to support the work. This includes the IAU Manager, PPI Co-ordinator, IAU Administrator, and two PPI User Support Workers. All research projects are coordinated by the PPI project coordinator with support from the PPI User Support Worker. Over the last 12 months the IAU has created a new role of Race Equality Ambassador, embedded into the team supporting wider race equality with local community groups to enhance engagement, in particular with Black African, Asian and Caribbean heritage communities.

The commissioned project was a cross-faculty study drawing on academic staff situated within Keele University’s School of Medicine and the School of Allied Health Professionals. The School of Allied Health Professions has a group of PPI contributors who advise on both staff and student projects in the field of neurology. PB has been an active PPI contributor for seven years and has extensive experience of working within research, helping to develop funding applications, including being a co-applicant and co-author. PB was, therefore, purposively selected to be one of the PPI contributors due to his extensive experience. With support from the PPI team within the School of Medicine, two other public contributors were identified. The NIHR suggest having at least two public contributors per project [11]. The process of inviting the public contributors was completed quickly so we could begin the project; if the PPI coordinator had more time they could have perhaps recruited additional public contributors.

The research team sought to involve experienced public contributors, who had been involved in research projects before. Whilst seeking experienced public contributors may not have been inclusive and limits perspectives from those less familiar with PPI, due to the short timeframe and allocated staff resources on the project, it was deemed to be important so the project could begin swiftly due to their baseline understanding of PPI and established partnership with Keele University.

### Aims of PPI within the commissioned project

The aim was to integrate PPI into four key stages of the commissioned project: when developing the research questions, when selecting the methods, interpreting the findings and when developing the recommendations.

### PPI activities

AM met with the PPI members on three occasions via Microsoft Teams. The content and purpose of the meetings were planned by AM and wider academic members of the research team. Within the first meeting the purpose of public involvement was jointly defined and a shared understanding of roles, responsibilities and expectations of public involvement was mapped out. The research questions, UK standards and Insights framework were discussed. Within the second meeting the study methods were further discussed and the initial findings were presented. Public contributors were shown how data from each report was mapped onto the UK Standards and Insights framework. Examples of discrepant data (e.g. data that the academic researchers deemed to fit into more than one UK Standard or Insight’s domain) were considered. The third meeting focused on how the findings had been modified in light of the previous meeting’s discussion, developing recommendations and discussing the PPI impacts upon this research project.

All meetings were audio-recorded. AM obtained permission to digitally record discussions for the purpose of clarity, and to enable her to write detailed summaries of the meetings for the research team to reflect upon. The audio-recordings were deleted once a summary of each meeting had been written. No verbatim quotations from public contributors were used in these summaries. Once a summary of each workshop was produced, they were sent via email to each public contributor who was invited to provide feedback on the summaries.

To capture how PPI influenced the commissioned project, AM documented how the project changed following each meeting. Within the meetings, public contributors discussed their views. After each meeting, AM liaised with academic members of the research team and discussed the feedback. Within the summary of each meeting, any changes to the project made as a result of feedback from public contributors were described.

### Reflections on the PPI activities

Once the project was complete, AM, AA and PB met virtually on several occasions to reflect on the PPI activities. The two other public contributors involved in the commissioned project were invited via email but did not respond, therefore, their reflections are not represented. In line with the recommendations from the commissioned project [8], we used the UK standards when reflecting upon our PPI activities and weave in relevant literature to discuss key reflections.

## Inclusive opportunities

Recent literature has questioned whose views are being represented within PPI activities and there has been a drive to make PPI an inclusive space in which people from different backgrounds can be involved equally [12]. Yet, PB noted that within this piece of work all public contributors were older white males and suggested that in the future researchers should seek to include those from under-served communities. All authors discussed that the building of relationships with under-served communities takes time, however, PB proposed that this aspect should have been factored into the allocated timeframe for the project. There is emerging evidence that the organisation of PPI in research can exclude people from lower socio-economic background and ethnic minorities [13]. Due to time and capacity restraints within this project, we could not fully involve the Race Equality Ambassador situated within the IAU. In future, we endeavour to include this individual to help to organise PPI activities and to enhance involvement with those from under-served communities.

Whilst all public contributors were offered payment, they had to formally register as a RUG member in order to receive remuneration. This could be a barrier to involvement for some public contributors who may not wish to be associated with a University, or may not easily navigate University systems and processes.

## Working together

The main challenge of involving public contributors within this project was time pressures. AM tried to carefully plan when to incorporate PPI activities within the six month timeframe. AM thought that allocating specific time to liaise with the wider research team to develop materials, meet with the public contributors and to write summaries was important. PB suggested that over-seeing PPI activities within a short time-frame requires operationally strong leadership.

Whilst PPI has been integral to this study, public contributors voiced that they did not feel that they could influence the research questions as these had already been set out by the NIHR. Previous literature has suggested that public contributors can help to identify research priorities which are relevant to them and formulate research questions [14]. When public contributors are involved in a commissioned piece of work, PB suggested that within all studies there needs to be clarity regarding the roles of public contributors within a study and what they can, and cannot, influence and that, ideally, the same public contributors should be involved throughout the research cycle.

AA and PB have established a long-term positive working relationship within an academic environment. PB has been involved in numerous research and education related public involvement activities. The other public contributors were also experienced in PPI activities. AM reflected that if a public contributor was unfamiliar with PPI they would have needed bespoke support to contribute to this project; something the timeframe did not permit.

The research team could not offer face-to-face meetings at the beginning of this project due to restrictions put in place during the COVID 19 pandemic. Whilst research has shown that throughout the pandemic there had been a reduction in the number of studies involving the public [15], we provided support for digital inclusion to mitigate this; as the project progressed, the University did begin to lift restrictions. An in-person meeting was offered for the third meeting, however, it was logistically challenging to arrange a meeting and transportation where all public contributors and researchers could attend (especially given we were nearing the end of the six month timeframe). Flexibility of meetings was key; researchers rearranged to meet public contributors’ needs and responsibilities, as appropriate.

PB suggested that maintaining a relationship with public contributors beyond the six month time-frame of this project was important so that they knew their input was not tokenistic. All three public contributors are now involved in an array of education and research activities within Keele University.

## Support and learning

Whilst public contributors could have sought advice from Keele University’s PPI co-ordinator or a User Support Worker, due to the time constraints of the project, researchers could not offer/develop a bespoke learning and support packaged for public contributors. A sharing or ‘hub’ of resources on how best to involve public contributors throughout the research cycle would have been beneficial [8]. PB suggested that, in the future, researchers may wish to ‘buddy’ a new public contributor with an experienced public contributor to help orientate them within activities.

## Communications

On reflection, developing a communications plan for PPI activities would have been beneficial; this plan could have included how to engage with under-served groups. Ideally, the content of the communications plan would have been co-produced with public contributors. Nonetheless, PB suggested that he still perceived that he had the potential to influence the project, particularly the evaluation tools used (UK Standards and Insights) and data analysis, within the meetings with AM; this was the way in which PB was used to being involved within PPI activities.

AM found describing the UK Standards and Insights Framework in lay language challenging; additional time was needed to do this. Next time AM is presented with such challenging content, she may discuss how to present it to public contributors with the Project Co-ordinator based within the IAU. The context of the project, and of NIHR research centres, was also complex. A number of acronyms were used; PB had to remind researchers on a number of occasions to explain what these meant. Projects may look to develop an abbreviation list which details each acronym and what they mean to each public contributor before the start of a project.

To ensure that the public contributors felt appreciated, AM allocated time before, during and after each meeting to liaise with public contributors on an informal level. During this time public contributors could raise any concerns or provide further feedback. After each meeting AM gave each public contributor her contact details and encouraged them to provide additional comments. AM suggested that whilst no public contributors contacted her following the meetings, it was important to show that she valued their contributions and she believed this created a positive dynamic between herself and the public contributors.

## Impact

The public contributors could not influence the research questions, but did influence the methods and findings of the commissioned project.

### PPI impact on the research process

#### Developing the research questions

Whilst the scope of the research (e.g. to review PPI sections of annual reports) had already been developed and commissioned by the NIHR, the researchers worked with the public contributors to re-word and refine the research questions. Previous research has drawn attention to the juxtaposition of inviting public contributors to use their voice but researchers or organisations retaining control over what can be changed as a result [16]. Within the PPI activities of the commissioned project there was a need to be transparent of how and what can be changed in response to PPI activities.

#### Developing the methods

When developing the methods, the researchers discussed the UK Standards. Whilst the UK Standards had been co-produced with public contributors, we further refined them and produced working definitions of each standard with the PPI group (please see Table 1). Illustrative exemplars of modifications and where the UK standards could be made more specific are underlined. The public contributors particularly thought that the ‘Communications’ standard needed to be expanded to include having processes to gather feedback and reflections, and could also include the sharing of learnings from PPI activities. The definitions of ‘Inclusive opportunities’ and ‘Working together’ could be expanded to include how these standards could be met on a strategic level.

**Table 1:** Definitions of the UK standards for public involvement

	Inclusive opportunities	Working together	Support and learning	Communications	Impact	Governance
**Original definition**	Offer public involvement opportunities that are accessible and that reach people and groups according to research needs.	Work together in a way that values all contributions, and that builds and sustains mutually respectful and productive relationships.	Offer and promote support and learning opportunities that build confidence and skills for public involvement in research.	Use plain language for well-timed and relevant communications, as part of involvement plans and activities.	Seek improvement by identifying and sharing the difference that public involvement makes to research.	Involve the public in research management, regulation, leadership and decision-making.
**Working definitions of the UK Standards**	To offer public involvement opportunities in the spirit of equality and diversity according to the research or strategic needs.	Work together with public contributors in a way that values all contributions on both a research and strategic level in a mutually productive way. Work with other organisations to identify and share PPIE best practice.	To identify training needs and to offer and promote support and learning opportunities that build both staff and public contributors’ confidence and skills for public involvement in research and strategy.	Use plain language for well-timed and relevant communications, as part of involvement plans and activities. To have processes in place to gather feedback and reflect upon PPIE activities. To share the learnings from patient involvement.	Seek improvement by identifying and sharing how public involvement has influenced public contributors and research and PPIE practice at a regional and national level. Understand the changes, benefits and learning gained from the insights and experiences of patients, carers and the public.	Involve the public in research management, regulation, leadership and decision-making. Provide the necessary resources and infrastructure to support PPIE activities.

The differences that the public contributors made to the standards are underlined

The public contributors also suggested that using the Insights framework would be an appropriate way to recognise varying levels of quality PPI activities, especially as the framework had been co-produced between academics and public contributors.

#### Interpreting the findings

One of the main challenges noted by AM was identifying the most appropriate way to generate public contributors’ perspectives on the qualitative data. Other researchers have trained public contributors in qualitative research methods, which was time-consuming and labour intensive [17]; both resources were scarce in this project. AM suggested more research or guidance is needed which focuses on the best ways to integrate PPI activities into qualitative data analysis.

AM gave a short overview of the qualitative analysis methods used. PB suggested that it is unrealistic to expect PPI members to develop complex analysis skills within one PPI meeting. One public contributor had a basic knowledge of qualitative analysis methods, the other two members had no knowledge of such methods. AM did not want any public contributors to feel like their contributions were inadequate because they were not knowledgeable about analysis methods. The brief overview of the analysis methods was important to address the power balance between public contributors with varying degrees of knowledge [18]. PB stated that a brief overview provided sufficient information for the public contributors to feel confident when contributing to analysis discussions.

The academic members of the research team agreed that whole, anonymous, reports would not be provided to public contributors to be analysed. We believed that reading these reports may have been burdensome for public contributors, yet this was arguably presumptive on the part of the research team. In the future, public contributors could be asked if they would like to read the whole report or whether they felt it may be burdensome, giving them a choice. AM did provide a brief overview of the findings (including quotations and a table showing how some data mapped onto the UK Standards and Insights framework), and presented any discrepant data (e.g., data whereby the academic researchers were unsure of which UK Standard/ Insights domain it should be mapped onto). When invited to offer their perspectives on quotations taken from reports, and which UK Standard/ Insights framework domain it mapped onto, this sometimes triggered public contributors to discuss their own experiences of PPI activities. The value of having public contributor’s perspectives was that each individual had their own lived experience of a PPI activities, the challenge was facilitating a discussion on the study’s findings which did not become side-tracked with the personal narratives of public contributors which lost sight of the data.

Public contributors’ perspectives on the data did not differ from the interpretation presented to them. Although this arguably enhances the trustworthiness of the findings, AM thought that it could have also been due to power dynamics. Public contributors may not have challenged the interpretation presented to them due to the perception that they did not have enough power, or experience in qualitative data analysis methods, to influence the analysis [19]. Public contributors were invited to share their perspectives on the findings and added to the interpretation offered by academic researchers.

#### Developing the recommendations for the commissioned project

Initially, recommendations for the commissioned project were developed for researchers, senior research leads, award-holders and the NIHR. Public contributors suggested that specific recommendations were needed which focused on what *they* could contribute. Following this, recommendations for public contributors were developed (e.g. to continue to promote the use of the UK Standards [8]). PB suggested that developing recommendations for public contributors shows that they are seen as equal stakeholders.

### Evaluation of PPI practice

A range of methods are available to evaluate PPI in research, often chosen based on the intended outcomes of the research and the time frame available. These approaches range in simplicity, from preparing an ‘impact log’ on the outcomes of the PPI [20], reflecting on the process of PPI [21], using the Cube evaluation framework [22] to the more comprehensive Public Involvement Impact Assessment Framework [23] or a Realist Evaluation [24].

Whilst impact was captured within written summaries and this reflective piece, we did not use or develop a specific framework for evaluating PPI activities. PB suggested that if the time constraints for the commissioned project had not been so tight, he would have liked to co-produce an evaluation framework with the researchers.

## Governance

Public voices were heard, valued and respected in all decisions; this is evidenced through the impact that PPI has had upon the project. Ideally, we would have liked public contributors to join monthly meetings with the NIHR to discuss the project, but this was not feasible due to an inability to find a time that would suit all. Within meetings with the NIHR, PPI was a standard item on the agenda. AM would discuss PPI activities and provide any feedback from the public contributors about the project. PB discussed that PPI plans for this project were regularly monitored and reviewed to see if we had met the four PPI aims of involvement when developing the research questions, selecting the methods, interpreting the findings and when developing the recommendations for the commissioned project. AA suggested that there was also visible and accountable responsibility for public involvement throughout the organisation through the role of a PPI co-ordinator.

## Co-produced recommendations

We have co-produced recommendations for researchers and research commissioners (please see Table 2).

**Table 2:** Co-produced recommendations

For researchers and staff working within PPI
1) Develop a shared understanding at the start of the project regarding the roles and responsibilities of each public contributor and researcher, along with what can, and what cannot, be changed in relation to a commissioned piece of research.
2) Ask public contributors their knowledge of qualitative methods and analysis and what, or if, they would like to learn about the particular method used; thus offering them a choice of how and when to be involved.
3) Allow time and space for personal reflections for both the researchers and public contributors throughout the project.
4) Creating and sharing a pool of resources (e.g. webinars, podcasts, written materials, videos, animations) related to involving public contributors throughout the research process.
5) Use evaluation frameworks as a basis for co-designing how we understand, refine and implement such frameworks for the evaluation of PPI.
For commissioners of research
1) To try to involve and invite the same public contributors throughout the research cycle, including deciding on what projects should be commissioned and developing research questions to the interpretation of findings; this may include public contributors working with both the commissioners of a project and the researchers conducting the project.
2) Provide adequate time within a project for meaningful PPI, which may include time to build relationships with public members from under-served communities and/or those with little knowledge about PPI.
3) To commission work which evaluates the best ways to integrate PPI activities into qualitative data analysis.

### Reflections on the process of writing this commentary

PB suggested that a strength of this co-produced commentary is a well-rounded understanding and reflection of PPI activities within a commissioned project with a short time-frame; by talking to each other we have created new knowledge in the form of the co-produced recommendations. All authors of this commentary found the process satisfying and enjoyable. PB suggested that whilst he enjoyed the conversational style of capturing reflections, as suggested by Staley and Barron [7], it was time-intensive and researchers may need a more formal evaluation method (such as the Cube evaluation framework [22]) to capture other public contributors’ experiences.

## Conclusion

We have written this commentary to create a culture of learning and sharing to enhance PPI practice. Key topics of reflection were: how difficult it is, in practice, to incorporate PPI into all aspects of the research cycle, especially when completing a commissioned research project on a short time-frame, and the complexities of incorporating PPI into qualitative analysis. We hope that the ‘co-produced recommendations’ can be used by other teams who wish to engage public contributors in research.

## Data Availability

No datasets were generated or analysed during the current study.

## Abbreviations

IAU: Impact Accelerator Unit
NIHR: The National Institute for Health and care Research
PPI: Patient and Public Involvement
RUG: Research User Group
UK: United Kingdom
